# Comparison of plants used for skin and stomach problems in Trinidad and Tobago with Asian ethnomedicine

**DOI:** 10.1186/1746-4269-3-3

**Published:** 2007-01-05

**Authors:** Cheryl Lans

**Affiliations:** 1 BCICS, University of Victoria, British Columbia, V8W 2Y2, Canada

## Abstract

This paper provides a preliminary evaluation of fifty-eight ethnomedicinal plants used in Trinidad and Tobago for skin problems, stomach problems, pain and internal parasites for safety and possible efficacy. Thirty respondents, ten of whom were male were interviewed from September 1996 to September 2000 on medicinal plant use for health problems. The respondents were obtained by snowball sampling, and were found in thirteen different sites, 12 in Trinidad and one in Tobago. The uses are compared to those current in Asia. *Bambusa vulgaris*, *Bidens alba*, *Jatropha curcas*, *Neurolaena lobata*, *Peperomia rotundifolia *and *Phyllanthus urinaria *are possibly efficacous for stomach problems, pain and internal parasites. Further scientific study of these plants is warranted.

## Background

Trinidad and Tobago is one country consisting of two adjacent islands located just northeast of the Venezuelan coast with a combined area of 5070 km^2 ^[1]. The human population of 1.25 million is multi-ethnic, multi-religious and multicultural and increases at 1% annually. In Trinidad, the major population centres are concentrated along the west coast and along an east-west transportation corridor in the north of the island [1].

The multi-ethnic population of Trinidad and Tobago is reflected in its folk medicinal use. Previous research has indicated that the folk medicines used by hunters are derived from ancient Amerindian practices [2]. This paper will continue to explore the cultural origins of Caribbean folk medicine by investigating the contribution of the Chinese to Caribbean folk medicine. Chinese medicine has been described as a complex and holistic system of medical practice with its own philosophy, diagnosis, treatment systems and pharmacology which also includes acupuncture, moxibustion and *Qi Gong*. However in this paper I will focus on 'Ben Cao' (Herbalism) [3].

The Chinese were the first Asian immigrants, arriving before the original East Indians who arrived in 1845. Chinese Tartars (192 men and one woman) were brought to Trinidad in the fall of 1806. These men from Macao, Penang and Canton were brought to cultivate tea but most were dissatisfied with local conditions and returned on the same ship [4,5]. The twenty-three who stayed made a living as entrepreneurs (butchers, shopkeepers, carpenters and market gardeners) and creolised (integrated into the local population).

Prominent sugarcane planters believed that the emancipation of Caribbean slaves in 1838 would create a labour shortage. In the 1840s, the British "opened" a labor market of displaced or impoverished peasantry in southern China to fill this shortage and 2,500 mainly-male Chinese were brought legitimately to Trinidad as indentured workers, or were 'shanghaied' (abducted by European traders) [6]. After the first Opium War (1840–42), and second Opium War, the British (as well as French and Americans) occupied twelve major ports (and colonized Hong Kong) [6]. China's defeats in the Opium Wars led to the deregulation of Chinese immigration. This combined with the unrest, rebellion, and war in China, facilitated the organized labour traffic of one million southern Chinese to the West from 1840 to 1875 [6].

Three vessels brought 1,100 Chinese indentured labourers to Trinidad in 1853 and 600 more came in 1865 and 1866. In 1862, 467 immigrants came from Hong Kong. Most of the immigrants arriving between1853 and 1866 came from the southern Guangdongprovince (Macao, Hong Kong and Canton). In the last 5 trips, a total of 2837 emigrants came from Macao, Amoy, Canton and Hong Kong. Chinese migration after 1911wasdriven by the Chinese revolution. Punti traders described Hakka prisoners as pigs on the bills of lading and shipped them to the Caribbean and South America [4,5]. Between 1920s and 1940s new immigrantsconsisted of the families and friends of earlier migrants. They came as merchants, peddlers, traders and shopkeepers, not indentured labour [4]. Almost 9,000 more Chinese immigrants came voluntarily from British Guyana to Trinidad over the next century, after having served their indentureship [5]. Chinese people now constitute approximately 1% of the Trinidad and Tobago population as an ethnic group but are also present in the large mixed-raced population of 18 – 25%.

There is one publication that describes the use of medicinal plants by the Chinese community in Trinidad [7]; it contained no plants in common with those in this research [1]. Nevertheless in the discussion section of this paper, comparisons will be made of the uses of the plants in Trinidad and Tobago and those current in Asia and South-east Asia. The ethnomedicinal literature available from Asia will be used in the non-experimental validation.

Fifty-eight plants used in ethnomedicine in Trinidad and Tobago for skin problems, stomach problems, pain and internal parasites are described in this paper and a non-experimental validation of them is presented. The recent publication of high-quality studies and clinical trials on the ethnomedicinal plants in this paper has enhanced the non-experimental validation of the plants presented in the discussion section.

## Methods

### Study design

This study adhered to the research guidelines and ethical protocols of Wageningen University in the Netherlands. Thirty respondents, ten of whom were male were interviewed from September 1996 to September 2000. The respondents were obtained by snowball sampling, and were found in thirteen different sites, 12 in Trinidad and one in Tobago. Snowball sampling was used because there was no other means of identifying respondents. The chief objective of the sampling method was to identify knowledgeable respondents.

Twenty respondents were interviewed once, the other ten (who were healers) were interviewed three or four times. Healers were also asked to reconstruct the circumstances and contexts of the plant uses so that the means of administration of the plants could be identified. No interview schedule of questions was used but a more qualitative, conversational technique. Plants were collected when available to verify that the common names used by each respondent were the same in each ethnic group as those recorded in the literature. The majority of the plants were identified at the Herbarium of the University of the West Indies but voucher samples were not deposited. This ethnomedicinal study was part of a larger research project on ethnoveterinary medicine; other data collecting techniques were used in the larger study [1].

### Non-experimental validation

The ethnomedicinal plants used in Trinidad and Tobago for skin problems, stomach problems, pain and internal parasites are presented in Tables 1 and 2.

**Table 1:** Ethnomedicinal plants used for skin problems in Trinidad and Tobago

	Scientific name	Family	Common name	Plant part used	Use
1	*Achyranthes indica*	Amaranthaceae	Man better man		Skin problems
2	*Acnistus arborescens*	Solanaceae	Wild tobacco	Leaves	Bathe babies for eczema
3	*Azadirachta indica*	Meliaceae	Neem	Leaves	Measles
4	*Bidens alba/Bidens pilosa*	Asteraceae	Needle grass/Railway daisy	Leafy branch	Bathe children
5	*Cassia alata*	Fabaceae- Caesalpiniaceae	Senna	Leaves	Skin problems
6	*Chamaesyce hirta/hypericifolia*	Euphorbiaceae	Malomay	Flower	Skin rashes, measles
7	*Croton gossypifolius*	Euphorbiaceae	Blood bush/Bois sang	Leaves	Bathe babies for eczema
8	*Eclipta prostrata*	Asteraceae	Congolala		Bathe for children's malnutrition for 9 days & woodlice nest
9	*Manihot esculenta*	Euphorbiaceae	Cassava	Leaves	Bathe babies for eczema
10	*Origanum vulgare*	Lamiaceae	Majoram		Bathe babies
11	*Sida carpinifolia *(syn. *Sida acuta*)	Malvaceae	Garaba broom	Leaf	Eczema
12	*Solanum americanum*	Solanaceae	Agouma, gouma	Plant	Bathe for children's malnutrition
13	*Spondias mombin*	Anacardiaceae	Hogplum	Leaves	Eczema

**Table 2:** Plants used for stomach problems, pain and internal parasites in Trinidad and Tobago

	Scientific name	Family	Common name	Part used	Use
1.	*Abelmoschus moschatus*	Malvaceae	Gumbo musque	Seeds	Grind in rum for foot cramp
2.	*Aframomum melegueta*	Zingiberaceae	Guinea pepper	Seeds	Carminative
3.	*Ambrosia cumanenesis*	Asteraceae	Altamis	Bark	Stomach pain, 2*3 inch piece bark in urine for 3 days use to wash foot for 3 days for arthritis
4.	*Aristolochia rugosa*,*trilobata*	Aristolochiaceae	Mat root, anico	Root	Stomach pain, colic, poisoning
5.	*Bambusa vulgaris*	Poaceae	Bamboo	Leaves	Poultice
6.	*Bidens alba/Bidens pilosa*	Asteraceae	Needle grass	Leafy branch	Cuts
7.	*Bixa orellana*	Bixaceae	Roucou	Root	Dropsy
8.	*Brownea latifolia*	Fabaceae	Cooper hoop	Flower, leaves	Gripe, pain
9.	*Cajanus cajan*	Fabaceae	Pigeon pea	Leaves	Food poisoning, colic, constipation
10.	*Capraria biflora*	Scrophulariaceae	Du thé pays	Leaves	Flavour for purgative
11.	*Cecropia peltata*	Cecropiaceae	Bois canôt	Stem	3 'Ridges' from inside stem boiled as a carminative
12.	*Centropogon cornutus*	Campanulaceae	Deer meat, crepe coq	Leaves	Snake, scorpion bite
13.	*Chamaesyce hirta*	Euphorbiaceae	Malomay		Diarrhoea
14.	*Citharexylum spinosum*	Verbenaceae	Bois côtelette	Leaf	Anthelmintic
15.	*Cocos nucifera*	Arecaceae	Coconut	Root- 7 inches, Shell	Dropsy, Hernia
16.	*Cola nitida*	Sterculiaceae	Obie seed	Seed	Any kind of pain
17.	*Cucurbita maxima*	Cucurbitaceae	Pumpkin	Seeds	Anthelmintic
18.	*Cucurbita pepo*	Cucurbitaceae	Pumpkin		Sprains, breaks
19.	*Dorstenia contrajerva*	Moraceae	Refriyau		Food poisoning
20.	*Eleusine indica*	Poaceae	Pied poule		Diarrhoea
21.	*Eupatorium macrophyllum*	Asteraceae	Z'herbe chatte		Pain
22.	*Eupatorium triplinerve*	Asteraceae	Ayapana, japanne	Leaves	Stomach problems (worms)
23.	*Ferula asafoetida*	Apiaceae	Asafoetida		Carminative
24.	*Jatropha curcas/gossypifolia*	Euphorbiaceae	White/Red Physic Nut	Leaf	Clean sores
25.	*Momordica charantia*	Cucurbitaceae	Caraaili	Vine	Stomach problems
26.	*Morinda citrifolia*	Rubiaceae	Noni	Leaves	Pains
27.	*Neurolaena lobata*	Asteraceae	Z'herbe á pique	Leaves	Tincture for arthritis
28.	*Nicotiana tabacum*	Solanaceae	Tobacco	Leaves	Arthritis
29.	*Nopalea cochinellifera*	Cactaceae	Rachette	Joint	Snake bites
30.	*Peperomia rotundifolia*	Piperaceae	Mowon		Diarrhoea
31.	*Petiveria alliacea*	Phytolaccaceae	Mapourite		Arthritis and rheumatism
32.	*Phyllanthus urinaria*	Euphorbiaceae	Red seed under leaf	Plant	Diarrhoea
33.	*Portulaca oleraceae*	Portulacaceae	Pussley	Plant	Anthelmintic
34.	*Punica granatum*	Punicaceae	Pome-granate	Seeds	Stomach problems
35.	*Rosmarinus officinalis*	Lamiaceae	Rosemary	Leaf	Arthritis, Snake bites
36.	*Scoparia dulcis*	Scrophulariaceae	Sweet broom	Root	Diarrhoea
37.	*Solanum melongena*	Solanaceae	Melongene	Fruit	Breaks
38.	*Tagetes patula*	Asteraceae	Marigold		Anthelmintic
39.	*Tamarindus indica*	Fabaceae	Tamarind		Scorpion bite
40.	*Tournefortia hirsutissima*	Boraginaceae	Chigger bush	Leaves	Tea, carminative, chiggers

The plant-based remedies were evaluated for safety and efficacy with a non-experimental method. Published sources such as journal articles and books and databases on pharmacology and ethnomedicine available on the Internet were searched to identify the plants' chemical compounds and clinically tested physiological effects. This data was incorporated with data on the reported folk uses, and their preparation and administration in Latin America, the Caribbean, Asia and Africa. For each species or genus the ethnomedicinal uses in other countries are given if available; then follows a summary of chemical constituents, in addition to active compounds if relevant (Tables 3 and 4). This type of ethnopharmacological review and evaluation has been previously published [2]. The plant uses in China are then given (Table 5) and a comparison of the uses in Trinidad and China is made in the discussion.

**Table 3:** Non-experimental validation of plants used for skin problems in Trinidad and Tobago

Scientific name	Validation	Reference
*Achyranthes aspera*	*Achyranthes bidentata *is a commonly used Chinese medicinal plant and is used in Nepal and in Mauritius and Rodrigues for skin diseases. *Achyranthes bidentata *polysaccharide can inhibit non-enzyme glycation in D-galactose induced mouse aging model *in vivo*. *Achyranthes aspera *leaf extract and the non-alkaloid fraction containing mainly non-polar compounds have chemo-preventive activity.	8–10
*Azadirachta indica*	A paste made of *Azadirachta indica *and *Curcuma longa *used to treat 814 people with scabies cured 97% of them within three to five days of treatment. *Azadirachta indica *(leaves, bark, fruit, flowers, oil, and gum) have the following properties: antimicrobial effects, *in vitro *antiviral activity, and antibacterial activity. Some active principles of *Azadirachta indica *are azadirachtin, salannin nimbin, and 6-desacetylnimbin. Clinical symptoms associated with toxocariasis in 1009 Trinidadian schoolchildren (aged 5–12 years) included eczema.	11–14
*Bidens pilosa*	*Bidens pilosa *is a commonly used traditional Chinese medicine.*Bidens pilosa *contains ethyl caffeate, a natural phenolic compound. Extracts of dried aerial parts of *Bidens pilosa *showed some antimicrobial activity as do components of the extract such as phenylheptatriyne, linolic acid and linolenic acid. The triterpenes as well as several flavonoids (aurones, chalcones) are antiinflammatory agents. The chloroform fractions from the roots of *Bidens aurea *are anti- parasitical *in vitro*. The constituents of *Bidens pilosa *explain the use of this plant in traditional medicine in the treatment of wounds, against inflammations and against bacterial infections of the gastrointestinal tract.	15–17
*Cassia alata*	"Jue ming zi" (*Cassia tora *L. and *Cassia occidentalis *L.) has traditionally been used to improve visual acuity and to remove "heat" from the liver in Chinese medicine. Modern physicians use "Jue ming zi" to treat hypercholesterolemia and hypertension. "Jue ming zi" contains chrysophenol, emodin, and rhein. Roasted "Jue ming zi" is given as a health drink tea. The antioxidant activity of the methanolic extracts of "Jue ming zi" (*Cassia tora *L. and *Cassia occidentalis *L.) was established. *Cassia alata *is used for skin problems in the Caribbean, India, in traditional East Asian medicine and in the Ivory Coast (West Africa) to treat bacterial infections caused by *Escherichia coli*, and fungal infections caused by *Candida albicans *and dermatophytes. *Cassia alata *L. possesses anti-inflammatory, analgesic, laxative and antiplatelet aggregating activity and it contains kaempferol-3-O-gentiobioside. *Cassia alata *has antifungal activity that may be attributed to chrysophanol. When *Cassia alata *extracts were evaluated relative to a standard antibacterial agent chloramphenicol and antifungal agent amphotericin B the extracts had therapeutic potential for the treatment of opportunistic infections of AIDS patients. A 10-year human study indicated that a *Cassia alata *leaf extract can be reliably used as a folk medicine to treat *Pityriasis versicolor*. The leaf extract contains anthraquinones, flavonoids, quinones and sterols and had no side-effects.	18–21
*Chamaesyce hirta*	*Chamaesyce hirta *is used in West Bengal for ringworm. Antibacterial effects of *Chamaesyce hirta *leaves were found by several investigators. An aqueous extract of *Chamaesyce hirta *strongly reduced the release of prostaglandins I2, E2, and D2. Additionally *Chamaesyce hirta *extracts exerted an inhibitory effect on platelet aggregation and depressed the formation of carrageenin-induced rat paw oedema.	22
*Croton gossypifolius*	*Croton cascarilloides *wood has been used historically to blacken teeth in Asia. *Croton cascarilloides *wood soot has limited antimicrobial activity against *Mutans streptococci*. Croton species are used in Thailand to treat dysmenorrhea, gastric ulcers, gastric cancers, and dysentery. *Croton kongensis *Gagnep., is known in Thailand as "Plao Ngeon" or "Plao Noi". A crude CH2Cl2 extract of *Croton *kongensis showed antimalarial and antimycobacterial activities. *Croton sylvaticus *showed 5-lipoxygenase inhibitory activity with IC(50) values <61 ppm. A review of papers published in 2003, found that *in vitro *and *in vivo *studies supported the use of *Croton lechleri *Mull. Arg. for wounds, tumors, herpes infection, the itching, pain and swelling of insect bites and other conditions.	23–26
*Eclipta prostrata*	*Eclipta prostrata *is commonly used as self medication by AIDS patients in southern Thailand and showed potential as a therapeutic agent against *Giardia intestinalis *infections. The hydroalcoholic extract of *Eclipta prostrata *plant showed antinociceptive, immunomodulatory and antiinflammatory effects.	27
*Origanum vulgare*	*Origanum *volatile oil has potential efficacy against the infection of dysentery bacteria (*Shigella sonne *(Sh. sonnei) and *Shigella flexneri*). The carvacrol constituent has the most effective antimicrobial activity in *Origanum vulgare*. Diarrheic children in Trinidad were positive for *Shigella *(33 or 14.0%), 4 for *Salmonella*, and 1 for Enteropathogenic *E. coli*. Two fecal samples were positive for *Campylobacter jejuni*, and 1 was positive for hookworm ova.	28–30
*Sida acuta*	*Sida acuta *contains ecdysterone, ephedrine, hentriacontane, hypolaetin-8-glucoside, beta sitosterol, stigmasterol and campesterol. These chemicals may be responsible for the plant's reported narcotic analgesic, anti-inflammatory and analgesic activity.	31
*Solanum americanum*	*Solanum americanum *extracts were active against *Microsporum gypseum *and *Cryptococcus neoformans *and showed intra-peritoneal subacute toxicity in mice. Alpha-solamargine isolated from the fresh fruits of *Solanum americanum *is a glycoalkaloid with biological activity against *Herpes simplex *I, *Herpes zoster *and *genital Herpes *and *Trypanosoma cruzi*. *Solanum melongena *contains an anthocyanin, delphinidin, which inhibits the collagenolytic ability of matrix metalloproteinases.	32, 33

**Table 4:** Non-experimental validation of plants used for stomach problems, pain and internal parasites in Trinidad and Tobago

Scientific name	Validation	#
*Aframomum melegueta*	A decoction of the leaves of *Aframomum melegueta *is used for rheumatism and as an anti-emetic agent and a decoction of the fruits for dysenteric conditions. The methanol extracts of the seeds were significantly active against Gram (+) and Gram (-) bacteria (*S*.*aureus, B*.*subtilis, E*.*coli, P*.*aeruginosa*) and fungi (*C. albicans, A*.*niger*). *Aframomum melegueta *has antimicrobial properties against *E. coli *and *Bacillus cereus*. The antioxidant extracts of *Aframomum melegueta *was attributed to its phenolic components. Scabies and acute poststreptococcal glomerulonephritis (the latter can be caused by several bacterial and viral infections) are frequently associated with *S. aureus *in Trinidad.	34–36
*Ambrosia cumanenesis*	The ambrosanolide-type sesquiterpene lactone cumanin (from *Ambrosia psilostachya*) showed a potent inhibitory effect in NO production (IC_50 _= 9.38 ± 0.38 μM) with low cytotoxicity.	37
*Aristolochia *species	The Chinese herb "Mu Tong" has included *Aristolochia manshuriensis *only since the 1950s. The classical Chinese herbal literature until the mid 17th century identifies "Mu Tong" as several *Akebia *species. From the 17th until the early 20th century "Mu Tong" was based on *Clematis *species. Renal failure due to ingestion of large doses of *Aristolochia manshuriensis *has been reported in China and other countries while no toxicity was recorded in traditional Chinese herbal texts. *Aristolochia's *topical anti-inflammatory activity has been recently described. Aristolochic acids, isolated from *Aristolochia longa *inhibited *Escherichia coli*, *Pseudomonas aeruginosa*, *Streptococcus faecalis*, *Staphylococcus aureus *and *Staphylococcus epidermidis*. The chloroform and hexane extracts of *Aristolochia trilobata *leaves and bark were active against *Escherichia coli *and *Pseudomonas aeruginosa *and *Staphylococcus aureus*.	38, 39
*Bambusa vulgaris*	The antiinflammatory effect of the methanol extract of the leaves of *Bambusa arundinacea *was significant when compared to standard drugs validating its use in Ayurvedic medicine. The methanol extract of *Bambusa arundinacea *also showed antihypersensitivity activity, immunosuppressive activity, wound healing property and antibacterial activity experimentally.	40
*Bidens pilosa*	The "Shidachuan" which was originally recorded in "Ben Cao Gang Mu Shi Yi" (A Supplement to the Compendium of Materia Medica) is "Longyacao" (*Agrimonia pilosa*). "Shijianchuan" should be "Guizhencao" (*Bidens bipinata*). Bioactive polyacetylenes were found in the methanolic extract of *Bidens pilosa *(whole plant). The antiinflammatory effect of aqueous extracts of the three plants *Bidens pilosa *var. *minor *(Blume) Sherff, *Bidens pilosa *and *Bidens chilensis *DC was significant. The immuno-suppressive activity of *Bidens pilosa *is attributed to the polyacetylene isolated from leaves. The water extract of *Bidens pilosa *showed a higher activity against *Bacillus cereus *and *Escherichia coli *than gentamycin sulphate. In one study diarrheic children in Trinidad were found to be positive for enteropathogenic *E. coli*.	41–43
*Bixa orellana*	*Bixa orellana *exhibited antimicrobial activity with a low MIC against *Escherichia coli *(0.8 microg/ml) compared to gentamycin sulfate (0.9 8 g/ml). *Bixa orellana *exhibited a better MIC against *Bacillus cereus *(0.2 microg/ml) than gentamycin sulfate (0.5 microg/ml).	43
*Cajanus cajan*	Extracts of roots and leaves of *Cajanus cajan *yielded 8 compounds: betulinic acid, biochanin A, cajanol, genistein and 2'-hydroxygenistein, longistylin A and C, and pinostrobin. The stilbenes, longistylin A and C, and betulinic acid showed moderate *in vitro *activity against chloroquine-sensitive *Plasmodium falciparum*. A protein was purified from the leaves and may enhance body immunosurveillance. *Cajanus indicus *protein possesses both a preventive and curative role against chloroform-induced hepatotoxicity and may act by an anti-oxidative defence mechanism.	44–46
*Capraria biflora*	The dried leaves of *Capraria biflora *(aqueous extract (50–200 mg kg(-1)) produced a moderate analgesic effect.	47
*Cecropia peltata*	*Cecropia pachystachya *has antioxidant properties. The two flavonoids orientin and iso-orientin, isolated from the active butanolic fraction could be responsible for the observed anxiolytic-like effect of *C. glazioui*. Steroids and amino acids in *C. peltata *may account for the antimicrobial activity exhibited against *E. coli*.	48, 49
*Centropogon cornutus*	*Centropogon cornutus *has a synonym *Lobelia cornuta*. Three new piperidine alkaloids were isolated from stems, leaves and flowers of *Lobelia laxiflora*. The residues obtained from the ethanol extracts from stems, leaves, and flowers showed anti-inflammatory protential.	50
*Chamaesyce hirta *syn. *Euphorbia hirta*	*Euphorbia hirta *aqueous extract is used for dysentery, colic, bronchial infections and to treat ulcers. The plant contains eucocyanidol, quercitol, camphol, quercetrin, dihydroellagitannins and dimeric hydrolysable tannins – euphorbins. Ethanolic extracts of the aerial parts of the plant showed antimicrobial activity against *Escherichia coli *(enteropathogen), *Proteus vulgaris*, *Pseudomonas aeruginosa *and *Staphylococcus aureus*.	51–53
*Citharexylum spinosum*	Six new iridoid glucosides and one known iridoid glucoside were isolated from the fruits and other parts of *Citharexylum caudatum*. The aerial parts of *Citharexylum spinosum *L., contain five iridoid glucosides, and one known lignan glucoside. When formulated in jojoba oil and applied to mice tails followed by infection with *Schistosoma mansoni cercariae*, the iridoid mixture from leaves of *Citharexylum quadrangular *blocked cercarial penetration and caused significant reduction (94%; P < 0.05) in worm burden in treated mice in comparison to controls.	54–56
*Cocos nucifera*	Coconut kernel fiber can protect cells from loss of oxidative capacity with the administration of the procarcinogen 1,2-dimethylhydrazine (DMH). The alcoholic extract of ripe dried coconut shell of *Cocus nucifera *showed antifungal activity against all dermatophytes tested with twice the concentration needed against *E. flocossum *(200 ug/ml).*Cocos nucifera *fruit exocarp has significant activity against all enteropathogens tested. All the strains tested were resistant to chloramphenicol; the two *Escherichia coli *species, the two *Shigella flexneri *species and the two *Salmonella *sp. species were sensitive to trimethoprim, and the two *Shigella sonnei *species were resistant. The authors concluded that coconut could be used as an alternative method to treat drug resistant enteric infections.	57, 58
*Cola nitida*	In Nigeria, *Cola accuminata*, *Cola nitida *and *Cola milleni *are used in ethnobotany for the treatment of diarrhea and dysentery.*Cola *species contain caffeine, koletein and kolatin alkaloids, proanthocyanin, magnesium, sodium, potassium bromide, cobalt, caesium, zinc and selenium. The *Mycobacterium bovis *was susceptible at 1000 μg/ml of methanol extract root bark of both *Cola nitida *and *Cola milleni *but insensitive to methanol extracts of both the leaves and stem-bark of the three *Cola *sp. tested. The MIC of the methanol root extract of *Cola nitida *against *Mycobacterium bovis *is 125 μg/ml. The MIC of methanol root extract of *Cola nitida *against the six ATCC strain of *Mycobacterium vaccae *ranged from 500 μg/ml to above 1000 μg/ml. The control Rifampicin is active against *M. bovis *at 5 μg and 10 μg/ml.	59
*Cucurbita *species	The minimum inhibitory concentration (MIC) of 23 gr. of pumpkin seed (+/- 73 seeds) (*Cucurbita maxima*) in 100 ml. distilled water as an antiparasitic agent using canine tapeworms with an intestinal isolation of 5 to 6 hours was determined. Alterations in helminthic motility were found at a dose of > 23 gr. There is a protheolithic effect with an average survival time of 38.4 minutes. The anthelmintic effect is increased at 30 and 32 gr.	60
*Dorstenia contrajerva*	*Dorstenia *species contain furanocoumarins with analgesic, anti-inflammatory, antibacterial, antiviral, anticoagulant, and photosensitizing activities. Prenylated chalcones are also found and may have anti-carcinogenic and antiproliferative properties. *Dorstenia contrajerva *was active toward *Giardia lamblia *with IC(50)<38 mug/ml. This antiprotozoal activity supports the popular use to treat diarrhoea and dysentery.	61–63
*Eleusine indica*	*Eleusine indica *ethanol extract showed activity against vesicular stomatitis virus. The plant contains hydrocyanic acid.	64
*Eupatorium macro-phyllum*	The ethanol extract of the leaves of *Eupatorium adenophorum *(100, 200 and 300 mg/kg, po) showed significant analgesic activity, compared to standard drugs diclofenac sodium and pentazocine Petroleum ether and methanolic extracts of leaves of *Eupatorium ayapana *showed broad spectrum antibacterial activity at the tested concentration (250–1000 μg/ml) except against *Shigella dysenteriae*. The petroleum ether extract also showed antifungal activity. Two extracts (dichloromethane and methanol), of the dried stems and leaves of *Eupatorium inulaefolium*, the S2 fraction of the hexane extract and neurolenin B from the dichloro-methane extract, showed statistically significant antiplasmodial activity	65–67
*Ferula *a *safoetida*	A *Ferula asafoetida *gum extract (3 mg/ml), decreased the average amplitude of spontaneous contractions of the isolated guinea-pig ileum to 54 +/- 7% of control. *Ferula asafoetida *gum extract (0.3–2.2 mg/100 g body weight) reduced the mean arterial blood pressure in anaesthetised rats.	68
*Jatropha curcas*	Two deoxypreussomerins were isolated from stems of *Jatropha curcas*. Two compounds had antibacterial constituents. *Jatropha curca*s crude bark extract accelerates the healing process of wounds on Wistar albino rats by increasing the skin breaking strength, granulation tissue breaking strength, wound contraction, dry granulation tissue weight and hydroxyproline levels. A significant decrease in epithelization period was also observed	69, 70
*Momordia charantia*	*Momordica charantia *may induce both intestinal and also systemic anti-inflammatory responses and may have antiviral activity.	71, 72
*Morinda citrifolia*	The lyophilised aqueous extract of roots of *Morinda citrifolia *produced a dose-related, central analgesic activity in mice. The analgesic efficacy of the Noni extract was less strong than morphine but non-addictive and had no side effects. *Morinda citrifolia *fruit powder demonstrated over 70% COX-1 inhibition. The extracts from *Morinda citrifolia *leaf (45%) showed moderate inhibition on COX-1. The extracts from *Morinda citrifolia *bark (27%) and *Morinda citrifolia *fresh fruit juice (38%) presented low inhibition on COX-1. The extract from *Morinda officinalis *root was inactive (9.87%) at a concentration of 3.4 mg/ml.	73–75
*Neurolaena lobata*	*Neurolaena lobata *has antinociceptive and antibacterial effects. When tested against *Brugia pahangi*, a lymphatic dwelling filarial worm, the ethanol extract of *Neurolaena lobata *showed potential macro- and micro-filaricidal activity.	76
*Nicotiana tabacum*	The lack of nicotine-induced analgesia assessed by the tail flick reflex test in female rats is consistent with human studies showing that nicotine reduces pain elicited by brief noxious cutaneous stimulation in male but not female subjects.	77
*Peperomia rotundifolia*	In south-east Asia, *Peperomia pellucida *is used for wounds, skin problems, abdominal pain and other pains and for headache. *Peperomia pellucida *is reported to have analgesic activity in mice, antibacterial activity against *Bacillus subtilis*, *Pseudomonas aeruginosa *and *Staphylococcus aureus*, and antifungal activity. *Peperomia pellucida *ethyl-acetate soluble extracts and crude methanolic extracts were active against Gram-positive and Gram-negative bacteria.	78
*Petiveria alliacea*	*Petiveria alliacea *extract showed an antinociceptive effect which account for its popular use as an analgesic. The oral administration of *Petiveria alliacea *root crude lyophilized extract at the highest dose of extract tested (43.9 mg/kg body wt.) significantly reduced the number of migrating neutrophils, mononuclear cells and eosinophils. The *Petiveria alliacea *root extract also showed a significant analgesic effect. Thiosulfinates, trisulfides and benzylsulfinic acid are antimicrobial compounds, with the benzyl-containing thiosulfinates having the broadest spectrum of antimicrobial activity.	79–81
*Portulaca oleracea*	The ingestion of purslane (*Portulaca oleracea*) leaves may have a protective effect against oxidative stress caused by vitamin A deficiency.	82
*Punica granatum*	*Punica granatum *was used by Egyptians in ancient times as a treatment for tapeworm and other parasites. A pomegranate extract at a low extract concentration (0.01% v/v) delayed bacterial growth of *Staphylococcus aureus *FRI 722, while a higher concentration (1% v/v) eliminated bacterial growth.	83
*Rosmarinus officinalis*	*Rosmarinus officinalis *has historically been used as ananalgesic and antirheumatic herb. The aqueous and ethanol extracts of *Rosmarinus officinalis *L. aerial parts induced a significant antinociceptive activity. In an observational study, a combination of reduced iso-alpha-acids from hops, rosemary extract and oleanolic acid decreased pain in patients suffering from rheumatic conditions and osteoarthritis.	84, 85
*Solanum melongena*	*Solanum melongena *contains significant quantities of histamine and serotonin.	86
*Scoparia dulcis*	*Scoparia dulcis *has traditionally been used to treat stomach troubles, inflammation, hemorrhoids, and hepatosis and as an analgesic. Biologically active substances from *Scoparia dulcis *include scoparic acid A, scoparic acid B, scopadulcic acid A and B, scopadulciol and scopadulin. The chloroform/methanol fractions *Scoparia dulcis *showed antimicrobial activity against the human pathogenic bacteria *Salmonella typhii*, *Staphylococcus aureus*, *Escherichia coli*, *Bacillus subtilis*, *Pseudomonas aeruginosa*, and *Proteus vulgaris *and the plant pathogenic fungi *Alternaria macrospora*, *Candida albicans*, *Aspergillus niger*, and *Fusarium oxysporum*.	87
*Tagetes patula*	*Tagetes erecta *callus cultures produce ascorbic acid as well as insecticidal pyrethrins.*Tagetes patula *oil contains several compounds with the major ones being limonene, (*Z*) and (*E*)-β-ocimene, dihydrotagetone, terpinolene, piperitone, peperitenone, *E *-caryophyllene and *trans *-sesquisabinene hydrate. The fourth instar larvae of *Aedes aegypti *(LC_50 _13.57, LC_90 _37.91) was most susceptible to *Tagetes patula *essential oil followed by *Anopheles stephensi *(LC_50 _12.08, LC_90 _57.62) and *Culex quinquefaciatus *(LC_50 _22.33, LC_90 _71.89).	88, 89
*Tamarindus indica*	In Thai traditional medicine, the fruit of *Tamarindus indica *is considered to be as a digestive, carminative, laxative, expectorant and a blood tonic. A crude *Tamarindus indica *seed extract extract inhibited the PLA2, protease, hyaluronidase, L-amino acid oxidase and 5'-nucleotidase enzyme activities of *Vipera russelli *venom in a dose-dependent manner. Mice that received the extract 10 min after the injection of venom were protected from venom-induced toxicity. The seed coat extract of *Tamarindus indica *has antioxidant activity. The extract is composed of flavonoids including tannins, polyphenols, anthocyanidin, and oligomeric proanthocyanidins. These flavonoids may produce vasorelaxant activity, increase capillary permeability and protection from oxidative stress. Excess nitric oxide production is associated with diseases such as autoimmunity, rheumatoid arthritis, inflammatory bowel disease and septic shock. *In vitro *studies demonstrated that the crude seed coat extract of *Tamarindus indica *suppressed nitric oxide production while producing no adverse effects.	90, 91
*Tournefortia hirsutissima*	In Taiwan, *Tournefortia sarmentosa *Lam. is used as a detoxicant, an antiinflammatory agent, and a circulation promoter to remove blood stasis. Alkaloids, flavones, triterpenoids, and cinnamates are found in the genus *Tournefortia*. The stems of *Tournefortia sarmentosa *contain five phenolic compounds as well as salicylic acid and allantoin. *Tournefortia rufo-sericeae *leaves contain pyrrolizidine alkaloids (5% of dry weight).	92, 93

**Table 5:** Chinese ethnomedicinal uses for the Chinese-origin plants or closely related species used in Trinidad

Trinidad ethnomedicinal plant	Chinese ethnomedicinal plant and practice
*Abelmoschus moschatus*	Geographical origin S.E. Asia. Myricetin a flavonol, is found in tea, berries, fruits, and the herb of *Abelmoschus moschatus*. This flavonol has both antioxidative and cytoprotective properties and has been used successfully to treat depression and anxiety in traditional Chinese medicine [94].
*Achyranthes aspera, Achyranthes indica*	*Achyranthes bidentata *is grown in the tropical parts of China, Korea and Vietnam. Its roots ("Niu Xi", Radix Achyranthes Bidentatae) are used in traditional Chinese medicine as a tonic, emmenagogue, antiarthritic, diuretic, and antifertility agent to nourish the liver and kidneys, strengthen bones and muscles and invigorate circulation [95].
*Aristolochia rugosa*,*A*. *trilobata*	The stem of *Aristolochia manshuriensis *(AMA, Guanmuton) is a traditional Chinese medicinal herb largely harvested from the Northeast of China. It is used as a diuretic, anti-inflammatory, to alleviate swelling and to treat rheumatism [96].
*Bidens alba/Bidens pilosa*	*Bidens parviflora *(Xiaohua-Guizhencao) is used as a traditional antipyretic, anti-inflammatory and anti-rheumatic medicine in China [97]. *Bidens pilosa *was introduced into Asia and is common in Taiwan.
*Cajanus cajan*	In Chinese folk medicine pigeon pea leaves are used to staunch blood, as an analgesic and to kill parasites [98].
*Cassia alata*	*Cassia obtusifolia *seed, called "Juemingzi", is used to treat eye infections, headache, and dizziness [99]. *Cassia alata *can be purchased in herb shops in Thailand.
*Croton gossypifolius*	There are 21 species of *Croton *distributed throughout the southern part of China. Several species including *C. kongensis *are used in traditional Chinese medicine to alleviate dysmenorrhea (fruits), as a purgative (seeds), and to treat dyspepsia (bark) and malaria (leaves) [100].
*Eclipta prostrata*	In Chinese medicine this plant is called "Eclipta Prostrata Herba" (Yetbadetajo Hert) [101]. It is also used in Taiwanese folk medicine.
*Eupatorium macrophyllum*	*Eupatorium chinense *grows in the south of China and is used for colds, snakebite and inflammation [102].
*Momordica charantia*	*Momordica charantia *seeds are known in Chinese medicine as "Ku guazi". They are used for infections and immune disorders [103].
*Morinda citrifolia*	Chinese traditional tonic herbal medicine "BaJiTian" (*Morinda officinalis*) has been prescribed in China for about two thousand years, for tonifying kidney, strengthening Yang-qi and relieving rheumatism [104].
*Phyllanthus urinaria*	*Phyllanthus urinaria *grows widely in China. It is used to treat jaundice, hepatitis B, neprolithiasis, and painful disorders [105].
*Portulaca oleraceae*	*Portulaca oleracea *(Ma-Chi-Xian), grows widely in China, and is used traditionally for alleviating pain and swelling. It has anti-bacterial, anti-viral, anti-diabetic, and immuno-modulating activity [106].
*Sida acuta*	This medicinal plant is named "Huanghuaren" [107].
*Tamarindus indica*	In Thai traditional medicine, the fruit of *T. indica *is used as a digestive, laxative, expectorant and blood tonic. The seeds of *T. indica *are used as an anthelmintic, antidiarrheal, and an emetic, and the seed coat is used to treat burns and aid in wound healing as well as against dysentery. [90], [91]

## Results

The ethnomedicinal plants used in Trinidad and Tobago for skin problems, stomach problems, pain and internal parasites are presented in Tables 1 and 2.

### Plants used for skin problems

Twelve plants are used for skin problems including one for the rash caused by measles plus one for shingles. The majority of the plants were being used for children including babies. The thirteen plants belong to nine plant families. Eight plants are used to bathe babies. *Acnistus arborescens Croton gossypifolius *and *Manihot esculenta *are used to bathe babies for eczema. *Bidens alba/Bidens pilosa *and *Origanum vulgare *are used to bathe babies and older children. *Eclipta prostrata *is combined with a non-plant material and used to bathe children for malnutrition. *Solanum americanum *is also used to bathe children for malnutrition.*Azadirachta indica *and *Chamaesyce hirta*/*hypericifolia *are used for measles. *Sida carpinifolia *(syn. *Sida acuta*) and *Spondias mombiin *are used for eczema. *Achyranthes indica*, *Cassia alata *and *Chamaesyce hirta/hypericifolia *are used for skin rashes and other skin problems.

### Plants used for stomach problems, pain, internal parasites

The medicinal plants used for stomach problems, injuries, endoparasites, arthritis and bites are combined in Table 2. This grouping partially reflects the analgesic activity of many of the plants used. Eighteen plants are used for stomach problems including diarrhoea. Another fifteen plants are used for various kinds of pain including cuts, bites, sprains and arthritis. Four plants are used as anthelmintics. Other plants in the table are used for dropsy. Twenty-seven plant families are represented in Table 2.

The following plants are used as carminatives: *Cecropia peltata*,*Aframomum melegueta*,*Ferula asafoetida *and *Tournefortia hirsutissima*.

The following plants are used for stomach problems: *Ambrosia cumanenesis*, *Aristolochia rugosa/trilobata*, *Capraria biflora, Dorstenia contrajerva*, *Cajanus cajan*, *Momordica charantia*,*Punica granatum*, *Brownea latifolia *and *Cocos nucifera*.

Diarrhoea is treated with the following plants: *Chamaesyce hirta*, *Eleusine indica*, *Peperomia rotundifolia*, *Phyllanthus urinaria *and *Scoparia dulcis*.

The plants used as anthelmintics are *Citharexylum spinosum*, *Cucurbita maxima*, *Portulaca oleraceae*,*Tagetes patula *and *Eupatorium triplinerve*.

Plants used specifically for pain are: *Brownea latifolia*,*Abelmoschus moschatus*, *Eupatorium macrophyllum*, *Morinda citrifolia *and *Cola nitida*.

Arthritis is treated with the following plants: *Nicotiana tabacum*, *Petiveria alliacea*, *Rosmarinus officinalis *and *Neurolaena lobata*.

Plants used for cuts, injuries and swellings are: *Solanum melongena*, *Jatropha curcas/gossypifolia*, *Bidens alba/Bidens pilosa*, *Cucurbita pepo*, *Tournefortia hirsutissima*, *Bambusa vulgaris*, *Bixa orellana *and *Cocos nucifera*.

Scorpion and snake bites are treated with *Tamarindus indica*, *Nopalea cochinellifera*, *Centropogon cornutus *and *Rosmarinus officinalis*.

### Non-experimental validation of plants used for skin problems in Trinidad and Tobago

For each species or genus the ethnomedicinal uses in other countries, particularly Asian countries, are given if available; then follows a summary of chemical constituents, in addition to active compounds if relevant to the condition being treated (Tables 3 and 4).

### Comparative evaluation of plants used for skin problems, stomach problems, pain and internal parasites

Table 5 contains a preliminary listing of the ethnomedicinal plants discussed in this paper that are used similarly in Chinese ethnomedicine. If the specific plant was not found in the literature search the closely related species that are used similarly in Chinese traditional medicine are listed.

The commonalities between Chinese traditional medicine and Trinidad and Tobago "bush medicine" are provided below.

*Abelmoschus moschatus *is used to treat depression and anxiety in traditional Chinese medicine [94]. In Trinidad and Tobago it is used for pain.

*Achyranthes bidentata *("Niu Xi" in Chinese medicine, Radix Achyranthes Bidentatae) is used as a tonic, to nourish the liver and kidneys, and invigorate circulation [95].*Achyranthes indica *is used in Trinidad and Tobago for skin rashes and other skin problems.

*Aristolochia manshuriensis *(AMA, "Guanmuton") is used in China as a diuretic and anti-inflammatory [96]. *Aristolochia rugosa/trilobata *are used in Trinidad and Tobago for stomach problems. Zhu claims that the Chinese herb "Mu Tong" has been based on *Aristolochia manshuriensis *only since the 1950s. The classical Chinese herbal literature until the mid 17th century identifies "Mu Tong" as several *Akebia *species and no toxicity related to "Mu Tong" was recorded in these traditional Chinese herbal texts.

*Bidens parviflora *("Xiaohua-Guizhencao") is used as a traditional antipyretic, anti-inflammatory and anti-rheumatic medicine in China [97]. Plants used for cuts, injuries and swellings in Trinidad and Tobago include *Bidens alba/Bidens pilosa*.

During the ethnomedicinal research one of the respondents claimed that the use of *Cajanus cajan *for internal parasites was a recent addition to Trinidad folk medicine. This ethnomedicinal practice in Trinidad is the same as that reported for the folk medicine of China (to kill parasites) [98] but no definitive statements about its origins can be made at this time. *Momordica charantia *seeds or "Ku guazi" are used for infections and immune disorders [103]; in Trinidad and Tobago the plant is used for stomach problems.

"BaJiTian" (*Morinda officinalis*) has been prescribed in China for about two thousand years, for tonifying the kidney, strengthening "Yang-qi" and relieving rheumatism [104]. Plants used for pain in Trinidad and Tobago include *Morinda citrifolia*.

*Phyllanthus urinaria *is extensively grown in China. It is used to treat jaundice, hepatitis B, neprolithiasis, and painful disorders [106]. Diarrhoea is treated with *Phyllanthus urinaria *in Trinidad and Tobago.

*Portulaca oleracea *("Ma-Chi-Xian") is grown widely in China, and is used traditionally for alleviating pain and swelling [106]. It is used as an anthelmintic in Trinidad and Tobago.*Tamarindus indica *fruit is used as a blood tonic and the seed coat of *Tamarindus indica *is used to treat burns and aid in wound healing in China. In Trinidad and Tobago, scorpion and snake bites are treated with *Tamarindus indica*.

## Discussion and conclusion

Vincent Yáñes, the captain of the caravel Niña reportedly dug up *Morinda citrifolia *in Hispaniola on December 30, 1492 [1]; yet this plant was not considered special in Trinidad until the forces of globalisation made "Noni" ubiquitous as an "Australasian cure-all" and it was then sold on the streets of Trinidad by herbalists and other traders [1]. This story illustrates that since Caribbean folk medicine is a product of globalisation and colonisation, research into its origins and plant uses is complex. Attributing specific uses to Chinese folk medicine would necessitate access to the earliest Chinese herbals.

The ship that brought 467 Chinese men, women, and children (from an original 549) in 1862 was the first ship to bring Chinese women to Trinidad. In the last 5 voyages (1862–1866), of 367 females embarked, 309 landed. The immigrant gender imbalance may have affected the dissemination of Chinese folk medicine into the Caribbean culture. Two wars taking place in eastern China in 1862 facilitated the immigration or abduction of Hakka peoples to the Americas and presumably the Punti peoples came in the later stages of immigration [108,109]. If any of these original immigrants had expertise in Chinese plants, besides knowledge of opium, they did not widely advertise this under the British colonial administration.

It may be the case that the Chinese contribution to Caribbean folk medicine has formed part of its earliest foundation and its provenance is not remembered. Research on the Chinese contribution to Trinidad is complicated by the fact that many of the Hakka research population have lived up to their migratory reputation – moving on to North America. Language is also a barrier.

Cuba and other Caribbean countries have not adopted the model of China's barefoot doctors. Cuba's medical diplomacy and investment in biotechnology generates symbolic capital: intangible qualities (like honour, prestige, and reputation) which appear opposed to strictly economic interests, are in fact convertible back into material capital [110]. The Cuban policy is to demonstrate that its socialist state can provide a modern health care system and need not settle for small-scale technologies like traditional medicine [110]. In contrast it has been estimated that 80% of medications used in Chinese rural areas are derived from Chinese materia medica and related products. These products are economical and therefore provide important cost savings [2,111,112].

Similarly to the process taking place in the Caribbean, younger people in Taiwan have been moving away from Chinese medicines because work pressures force them to seek faster cures from allopathic doctors [2]. However tonic herbs such as "Danggui" (*Radix Angelica sinensis*), "Huangqi" (*Radix Astragali*/*Astragalus membranaceus*), "Gou Qi Zi" (*Fructus barbarum*) and "Renshen" (*Radix Panax ginseng */*Panax notoginseng*), are used by Taiwanese families in slow-cooking winter meals. These herbs are also popular for postnatal care, for the eldely and for postsurgical therapy [2].

Non-experimental validation is a new approach that is designed to introduce cost effectiveness into medicinal plant research. The findings of the non-experimental validation suggest that the majority of the therapeutic applications of the plants used in Caribbean folk medicine listed in this paper are justified, and more studies are warranted to explore their efficacy. All of the plants used in Trinidad and Tobago for skin problems merit clinical trials. The plants used for stomach problems, pain and internal parasites that should take priority in clinical trials are *Bambusa vulgaris*, *Bidens alba*, *Jatropha curcas*, *Neurolaena lobata*, *Peperomia rotundifolia *and *Phyllanthus urinaria*.

## Competing interests

The author(s) declare that they have no competing interests.
